# Evolved biocultural beings (who invented computers)

**DOI:** 10.3389/fpsyg.2015.01047

**Published:** 2015-07-22

**Authors:** Louise Barrett, Thomas V. Pollet, Gert Stulp

**Affiliations:** ^1^Department of Psychology, University of LethbridgeLethbridge, AB, Canada; ^2^Department of Social and Organizational Psychology, VU UniversityAmsterdam, Amsterdam, Netherlands; ^3^Department of Population Health, London School of Hygiene and Tropical MedicineLondon, UK

**Keywords:** evolutionary psychology, extended mind, cognition, culture, embodied cognition

## Introduction

Many thanks to Bryant (2015) for keeping the conversation lively, and engaging in further debate on our paper (Barrett et al., 2014). Although Bryant raises several interesting points, it appears that, as with our previous commentators, there was some misunderstanding of our aim, which simply was to answer the question posed for us: does evolutionary psychology represent an alternative to computational theories of mind? To reiterate, we suggested that Santa Barbara-style EP could not be an alternative given that it already is a computational theory of mind. Bryant (2015) apparently considers this question ill-posed, given his assertion that viewing “the mind as a case of digital computation” is “the only game in town.” Our friendly suggestion here is that perhaps he needs to get out a little more, and sample more fully the alternatives on offer.

## Not the only metaphor in town

“Freud often compared the brain to hydraulic and electro-magnetic systems. Leibniz compared it to a mill, and I am told the ancient Greeks thought the brain functions like a catapult. At present, obviously, the metaphor is the digital computer.”

~ John Searle

To assert that there is no alternative to digital computation is a philosophically weak position, not least because the history of science provides ample evidence of prominent ideas eventually shown to be wrong: phlogiston and the luminiferous aether were also “the only game in town” once upon a time. The brain has been likened to many other cultural tools that were, unsurprisingly, considered to be of great significance in their time; the computational metaphor is one in a long line of metaphors that reflect the most advanced technology of their day. There is no reason to imagine that we have finally managed to hit on the correct one, as opposed to the one that just reflects something about the times in which we live (Barrett, 2011).

Our main point, though, is that digital computation really isn't the only game in town. Among other work cited in our original paper, Chemero (2009) provides the most recent and comprehensive treatment of a non-computational-representational approach to mind (see also Anderson (2014, 2015) for a more neuroscientifically-focused account that similarly concludes the best way to understand the brain may be in enactive and ecological terms). Furthermore, this approach is not restricted to philosophical theorizing but also includes empirical work (see Dotov et al., 2010, as well as the examples given in Chemero, 2009). There have also been earlier incarnations of a non-representational approach to cognition (e.g., Rosch et al., 1992; Thelen and Smith, 1994; Kelso, 1997) along with Gibson's (1979) anti-representational theory of perception (see Barrett, 2011 for a review). Given Bryant's (2015) assertion of no scientific alternative to computation, he implies either that such genuinely non-computational-representational approaches do not qualify as science, or else he has misunderstood them. Although all we need do to counter Bryant's position is point to these scientifically credible alternatives, in what follows we consider briefly some of the arguments against a strong computational position. We then go on to discuss his other assertions regarding the relationship between culture and cognition.

It is telling that Bryant (2015) adopts a rather “Hegelian” approach in his commentary (see Chemero, 2009, and our original article), asserting the necessity of digital computation and information processing (conceived in terms of Shannon information theory: G. Bryant, Pers. Comm.), rather than providing arguments or evidence for it. As Wallace (2007) discusses, echoing Searle (1990) before him, the notion of the brain as engaged in digital computation is not a scientific discovery, like the moons of Saturn, but is instead a claim: a claim that looking at the brain in this particular way is useful. As Wallace (2007) argues, Shannon's theory was a pragmatic solution to an explicitly human engineering problem, and was never intended as a scientific theory of cognition. Using Shannon information to model human cognition is fundamentally flawed because, as Wallace (2007) discusses, human cognitive systems violate the assumptions under which Shannon information applies. Wallace (2007) further notes that use of terms like “information processing” to describe brain function can lead to a dualist position. Such thinking pervades Klasios's (2014) commentary, for example, when he argues that EP deals only with information processing and not neural activity as such, giving rise to the brain possessing two distinct qualities: the material activity of its neurons and, as Wallace (2007) calls it, the non-material, “mysterious world of information.”

Bryant's argument rests on the notion that adopting an information theoretic view is, in essence, a functionalist perspective, asking what role a given process plays, rather than the specific manner in which it is brought about or implemented. This is entirely reasonable—such a position has helped avoid a particular kind of “neural chauvinism” that suggests there is something inherently special about biological brains, so excluding any form of artificial intelligence from consideration. In addition, cognitive integration is often characterized as a form of “extended functionalism” (e.g., Wheeler, 2010) precisely because it attempts to expand the bounds of the cognitive system beyond the biological brain. That said, it is also apparent that understanding neural implementation is crucial to generating well-founded hypotheses about brain function. Neurobiological data are particularly useful for guiding evolutionary theories by constraining our hypotheses with respect to what brains can reasonably be expected to achieve (see Colombo, 2013 and Peters, 2013). Bryant and Klasios both stick to the classical cognitivist EP party line, justifying a strategy of studying the computational-algorithmic levels alone, but we think that EP, and evolutionary approaches more generally, would benefit from using neurobiological data to inform their theoretical stance, thus grounding our knowledge in living biological systems; we are, after all, attempting to explain how such living biological systems work. Such a stance is also a natural element of the embodied perspective we endorse, which argues that the way brains are put together will matter for cognition (that is, the way that neurons, glia, neurotransmitters and neuromodulators actually go about doing their job) and how adaptive behavior in the world is generated.

From our perspective, then, it seems well-worth considering the radical non-representational/non-computational alternative proposed by Chemero (2009), as well as related work on cognitive integration. As Chemero (2009) himself points out, and as we acknowledged in our original article, embodied/dynamical approaches do not entail a rejection of all representational theories of mind. Clark (1997), for example, argues that there are certain “representation-hungry” (i.e., linguistic) processes that do not seem amenable to an account grounded in coordinated sensorimotor processes alone. Thus, there is some effort being made to reconcile embodied and dynamical approaches with computational/ representational theories (e.g., Barsalou's, 1999 “perceptual symbol systems” and Clark's, 1997 “dynamic computationalism”). At a minimum, however, the recognition of alternatives to computational-representational theories of mind make it possible to ask some penetrating questions about the nature of representation in a computational model of mind, and whether such representations are, in fact, doing all of the cognitive work, all of the time (Barrett, 2011). Thus, when Bryant (2015) asserts that cognitive integration is a computational theory he is not wrong, but nor is he right. More specifically, the issue of whether cognition is extended can be viewed as orthogonal to whether cognition should be viewed as computation (see Sutton, 2014): the issue at stake is where the bounds of the cognitive system should be drawn, and whether bodily resources, material artifacts, and other aspects of the environment can be considered constitutive parts of the cognitive system or simply causally related to them. We argued for the constitutive approach, because this follows naturally from a radical embodied view that treats cognitive processes as products of the interaction between brain, body, and environment and not the brain alone (see also Chemero, 2009; Barrett, 2011; Hutto and Myin, 2013). Even if one wishes to adhere to a computational framework, however, cognitive integration can and does represent an alternative approach to standard cognitive psychology because it views cultural artifacts (in the human case) and other environmental resources as an integral part of cognitive systems (a point made in both Barrett et al., 2014, and reiterated in Stulp et al., 2015); cognitive integration does not view the brain alone as the part that does all the heavy-lifting. To counter this, as Bryant (2015) and Klasios (2014) do, simply by insisting that brains compute is to precisely miss this point.

## Loops, not arrows

Bryant (2015) also suggests that we have got our causal arrow largely pointing backwards. According to Bryant, culture is primarily shaped by our brains and bodies, and not vice versa (although he then goes onto suggest something very similar to our position where “the outputs of such processes feedback iteratively into an evolutionarily dynamic cultural knowledge system,” which points to a tension—if not an outright contradiction—in his argument). Of course, human brains are involved in the creation and use of artifacts and other forms of cultural representation, and hence brains must be involved in the shaping of culture—this is not at odds with anything we said in our original paper. The argument from the extended mind, however, is that, by extending our cognition beyond the biological brain, we become capable of feats that would otherwise be impossible. Malafouris' (2013) recent analysis of how physical artifacts allowed us to make the transition from numerosity to a formal concept of number and hence mathematics is another good example (see also Menary, 2007). Our position, then, is that we did not get the causal arrow backwards because there is no arrow. Instead, there are loops of continual reciprocal causation, with social activities and material culture both shaping and being shaped by the brain in an ongoing cycle.

By seizing on our example of timeliness as ultimately reflecting concerns about coordination and cooperation, Bryant (2015) over-simplifies and trivializes our position. Perhaps we made our point too flippantly. What we were attempting to convey was the idea that, through our invention of hours, minutes, and seconds, along with devices to measure their passing, our specific concept of time as a fourth dimension (and so on through to the concept of space-time that characterizes Einstein's theory of relativity), has fundamentally transformed aspects of human thought and practice. There seems no way that our use of time can be reduced entirely to the demands of social coordination and cooperation. Indeed, it is interesting to note that both Basu and Waymire (2006) and Mullins et al. (2013) make exactly the reverse argument to Bryant (2015) suggesting that large-scale human cooperation was dependent on material culture, namely writing and record-keeping (although these authors argue that this allowed us to “transcend” our evolved psychology, we would suggest this represents an example of how human psychology is inherently extensive and integrated with environmental and cultural resources). Although timeliness could be a by-product of social norms and customs as Bryant suggests, the concept of time is not: our invention of various ingenious ways to measure time and how we use these to shape our lives, permit us to go far beyond anything that our Pleistocene ancestors were capable of. Of course, humans are not “infinitely flexible and unconstrained by past selection,” but one has to admit there is something about the sheer inventiveness of modern human behavior that quite clearly reflects the manner in which cultural artifacts augment, enhance, and extend our evolved biological brains.

## Functional fuzziness

Bryant (2015) identifies one last failing on our part concerning the relationship between domain-generality and specificity, with reference to our argument on incest taboos. Namely, he suggests that we did not consider the possibility that unconscious mechanisms guide our behavior under such circumstances. Far from failing to acknowledge this, however, we cited Westermarck (1921) in precisely this context. It is odd that Bryant picks up on an aspect of our argument that was made explicit in our original piece, but ignores its substance, which was to counter the idea put forward by Cosmides and Tooby (1994) that incest avoidance requires innate domain-specific knowledge because such knowledge could not, even in principle, be learned. Instead, Bryant attempts to shift the emphasis, making the point that “just because a mechanism works across content domains, it is still functionally specialized. The scope of a mechanism is independent from whether it has design features.” To the extent that we understand this statement, it seems to promote a rather fuzzy notion of functional specialization, and deny the very motivation for EP-style “design thinking” in the first place: that is, the notion that specialized tasks require specialized mechanisms, and cannot be solved effectively by general-purpose mechanisms that apply across several domains. Such a statement also raises an empirical worry for, if it is true that a mechanism's scope is independent of its design features, how does one go about identifying evolved functionally-specialized mechanisms applied to a new domain as opposed to evolved domain-general mechanisms operating on one of the several tasks to which they are well-suited? There seems to be no means of distinguishing the two. Consequently, when Bryant asks: where is the argument here? We would suggest that, not only is there an argument to be had, but it is one that cuts to the very heart of the EP project.

## Why all the fuss?

Summing up, Bryant (2015) suggests the gap between human behavioral ecology and EP is closing, but notes that Symons (1987) question remains: if we're all Darwinians, what's all the fuss about? For us, the fuss is about the rather restrictive view of human psychology promoted by EP, and the failure of some of its assumptions to withstand close scrutiny; a topic that occupied two-thirds of our original paper. More broadly, we think it is worth considering whether we should continue to base our model of the mind on inanimate computation (a notion that has human intentionality built into it at source) or whether we should pursue a truly evolutionary route that grounds psychology (of both human and non-humans) in living biological systems—a view that further permits the study of humans as hybrid embodied-extended biocultural beings who invent all kinds of things and so continually reinvent themselves. For us, this is another good reason why some Darwinians should continue to make a fuss.

## Funding

LB is supported by NSERC Canada Research Chairs and Discovery Grant Programs; TP is supported by The Netherlands Organisation for Scientific Research (Veni scheme; 451.10.032); GS is supported by The Netherlands Organisation for Scientific Research (Rubicon Post-Doctoral Fellowship).

### Conflict of interest statement

The authors declare that the research was conducted in the absence of any commercial or financial relationships that could be construed as a potential conflict of interest.
